# P04.89. Utilization, interest and beliefs about complementary, alternative and integrative medicine: a survey of pediatric caregivers and healthcare providers

**DOI:** 10.1186/1472-6882-12-S1-P359

**Published:** 2012-06-12

**Authors:** N Vincent, M Boerner-Ott, J Moghal

**Affiliations:** 1 CHOC Children's, Orange, USA

## Purpose

There is limited information available about factors that influence interest, beliefs and utilization of CAIM (Complementary, Alternative, and Integrative Medicine) in a pediatric population. Thus, the current study examined these factors through questionnaires to both (1) caregivers of pediatric patients and (2) pediatric healthcare providers. This two-part study was conducted at CHOC Children’s, a 238-bed pediatric hospital in Orange, California.

## Methods

Similar but not identical surveys were administered to each of the two groups. Surveys were administered to pediatric patients’ caregivers (in both inpatient and outpatient settings) to assess CAIM interest, utilization and associated beliefs. This survey included demographic information and a questionnaire assessing use/interest in 31 different types of CAIM interventions. It also included the 11-item Holistic Complementary and Alternative Medicine Questionnaire (HCAMQ), assessing beliefs about the scientific validity of complementary and alternative medicine (CAM) and beliefs about holistic health. A slightly altered survey was administered to healthcare providers, to assess CAIM interest, utilization, and associated beliefs. This survey included questions about healthcare specialty and years of practice. It also included a similar questionnaire assessing referral to 31 different types of CAIM interventions. It also included the CHBQ - a 10-item Complementary and Alternative Medicine Health Belief Questionnaire, measuring attitudes and beliefs toward CAM.

## Results

Surveys were fully completed by 228 patients’ caregivers and 195 healthcare providers from the Orange County area. The ages of the children whose caregivers responded ranged from infancy to 21, with a diverse range of ethnic backgrounds represented. Among the 195 surveys completed by healthcare providers, 26 percent were completed by physicians, 14 percent by medical residents, 20 percent nurses, and the remainder from other ancillary fields (including psychologists, occupational and physical therapists and dieticians).

## Conclusion

Details of the study will be discussed in detail during the poster presentation, including utilization rates of various CAIM treatments, and factors associated with interest, use and beliefs about CAIM.

